# bioMIND‐A Novel Approach to Integrating Biomarkers in the Diagnostic Workup for Alzheimer's Disease

**DOI:** 10.1002/alz70860_103496

**Published:** 2025-12-23

**Authors:** Ameya Patwardhan, Kayla VanderPloeg, Adrian Budhram, Ting‐Yim Lee, Sarah Best, Michael J Borrie, Jaspreet Bhangu

**Affiliations:** ^1^ Lawson Research Institute; Parkwood Institute, London, ON, Canada; ^2^ Cognitive Clinical Research Group Parkwood Institute, London, ON, Canada; ^3^ Western Univerisity, London, ON, Canada; ^4^ Western University, London, ON, Canada; ^5^ Lawson Research Institute, London, ON, Canada; ^6^ Cognitive Clinical Research Group, Parkwood Institute, London, ON, Canada; ^7^ Schulich School of Medicine and Dentistry, Western University, London, ON, Canada; ^8^ Parkwood Institute, London, ON, Canada

## Abstract

**Background:**

Biomarkers for amyloid and tau pathology have revolutionized Alzheimer's Disease (AD) management, enabling pathological confirmation and therapeutic targets. Currently, there are no standardized care pathways for biomarker testing in Canada and most eligible patients are offered biomarker testing late, if ever, in the diagnostic pathway. The objective of this study was to investigate the feasibility of a “biomarker first” approach in the diagnostic pathway of AD in a real‐world cohort of patients.

**Method:**

This prospective, observational study enrolled patients with subjective cognitive decline, amnestic mild cognitive impairment (aMCI), or mild dementia awaiting assessment at a tertiary memory clinic. Standardized inclusion criteria were based on disease modifying therapy eligibility from referral information provided by a primary care physician. Participants underwent cognitive and functional testing, lumbar puncture for cerebrospinal fluid (CSF) collection, and amyloid PET scans prior to being evaluated by a specialist. Participants then underwent specialist clinical assessment with the results of their biomarker testing. A standard of care group (biomarker second) was included for comparison.

**Result:**

276 participants were screened with 47 (17.02%) eligible. Exclusions were due to age (10%), low MoCA scores (16%), medical comorbidities (15%), inability to complete study procedures (14%), insufficient referral information (16%), MRI contraindications (5%), other neurodegenerative diseases (5%), psychiatric disease (2%). 28% of eligible participants were reluctant to undergo lumbar puncture. Demographics and cognitive profiles were comparable between the two groups, with mean MMSE scores of 26.28 ±2.50 in the biomarker first group and 26.15 ±2.94 in the biomarker second group (*p* = 0.719). CSF biomarkers consistent with AD were detected in 78.5% of biomarker first group compared to 63.15% of biomarker second group. CSF results predicted positive amyloid PET scans in all patients.

**Conclusion:**

This study demonstrates that 17% of “real‐world” patients referred to a tertiary memory clinic would be eligible for biomarker testing based solely on screening information provided by primary care. The biomarker‐first approach is feasible and accurate, enabling earlier diagnostic clarification and personalized treatment opportunities for AD. This pathway could help shift care from late‐stage symptom management to early intervention targeting disease pathology, improving outcomes and optimizing healthcare delivery.

